# Current Self-Reported Symptoms of Attention Deficit/Hyperactivity Disorder Are Associated with Total Brain Volume in Healthy Adults

**DOI:** 10.1371/journal.pone.0031273

**Published:** 2012-02-10

**Authors:** Martine Hoogman, Mark Rijpkema, Luc Janss, Han Brunner, Guillen Fernandez, Jan Buitelaar, Barbara Franke, Alejandro Arias-Vásquez

**Affiliations:** 1 Department of Psychiatry, Radboud University Nijmegen Medical Centre, Donders Institute for Brain, Cognition and Behaviour, Nijmegen, The Netherlands; 2 Department of Human Genetics, Radbound University Nijmegen Medical Centre, Donders Institute for Brain, Cognition and Behaviour, Nijmegen, The Netherlands; 3 Centre for Cognitive Neuroimaging, Radboud University Nijmegen, Donders Institute for Brain, Cognition and Behaviour, Nijmegen, The Netherlands; 4 Department of Cognitive Neuroscience, Radboud University Nijmegen Medical Centre, Donders Institute for Brain, Cognition and Behaviour, Nijmegen, The Netherlands; Instituto de Ciencia de Materiales de Madrid - Instituto de Biomedicina de Valencia, Spain

## Abstract

**Background:**

Reduced total brain volume is a consistent finding in children with Attention Deficit/Hyperactivity Disorder (ADHD). In order to get a better understanding of the neurobiology of ADHD, we take the first step in studying the dimensionality of current self-reported adult ADHD symptoms, by looking at its relation with total brain volume.

**Methodology/Principal Findings:**

In a sample of 652 highly educated adults, the association between total brain volume, assessed with magnetic resonance imaging, and current number of self-reported ADHD symptoms was studied. The results showed an association between these self-reported ADHD symptoms and total brain volume. Post-hoc analysis revealed that the symptom domain of inattention had the strongest association with total brain volume. In addition, the threshold for impairment coincides with the threshold for brain volume reduction.

**Conclusions/Significance:**

This finding improves our understanding of the biological substrates of self-reported ADHD symptoms, and suggests total brain volume as a target intermediate phenotype for future gene-finding in ADHD.

## Introduction

Attention Deficit/Hyperactivity Disorder (ADHD) affects 1–4% of adults and has an even higher prevalence in children [1]. Structural magnetic resonance imaging (MRI) studies in children with ADHD have found reductions of around 3% in total brain volume [2] as well as in specific (sub-) cortical brain regions [3]. Brain volumetry studies in adult ADHD patients mainly reported reductions of brain volume in the prefrontal cortex [4], [5], caudate nucleus [6], [7] and amygdala [8], as well as a marginal increase of nucleus accumbens volume [4]. Only some of these findings have been replicated to date [7], [9].

Currently, ADHD is thought to be the extreme of a continuum of behavior in the population [10]. This was illustrated, for example, by work of Lubke *et al*, in which attention problems in children vary along a severity continuum from mild to severe; children with ADHD were at the extreme of the continuum [11]. Further evidence for dimensionality can be found in studies in which neurobiological characteristics associated with ADHD show evidence for dimensionality. The study of Shaw *et al* showed the reduced cortical thinning with increasing ADHD severity [12]. However these studies concern childhood ADHD, dimensionality of ADHD symptoms in adulthood has not been investigated in this regard.

In order to get a better understanding of the neurobiology of ADHD, we take the first step in studying the dimensionality of current adult ADHD symptoms, by investigating the association between a biological construct of ADHD, brain volume, and current self-reported ADHD symptoms in an adult population. In addition, ADHD is a highly heritable disorder [13] yet gene-finding approaches have been relatively unsuccessful in ADHD to date [14] potentially due to its diagnostic/phenotypic and genetic complexity, and given the high heritability of brain structure [15], this study might aid in characterizing total brain volume as a target intermediate phenotype to improve gene-finding.

## Materials and Methods

### Participants

In this study, 652 subjects aged 18–35 years from the Brain Imaging Genetics (BIG) study at the Donders Institute for Brain, Cognition and Behavior of the Radboud University Nijmegen Medical Centre were included. The BIG study is a study of self-reported healthy individuals included into earlier imaging studies at the Donders Centre for Cognitive Neuroimaging. Structural imaging data of these studies were pooled for which subjects had to give their consent. About 80% of the subjects have their consent to include their structural imaging data. Subjects were of European Caucasian descent and generally highly educated (more information on inclusion of subjects see [16]). The study was approved by the medical ethical committee (CMO regio Arnhem/Nijmegen) and all participants provided written informed consent prior to participation.

### Behavioral measures

Assessment of ADHD symptoms was performed through internet-based testing, as part of an electronic questionnaire and test battery. Subjects were asked to complete the ADHD DSM-IV-TR Rating Scale for current symptoms in adults [17]. Symptoms were reported over the last 6 months. Participants had to answer 23 questions on a 4-point scale (never, sometimes, often, very often). The 23 current item scores were recalculated to the original 18 DSM-IV-TR ADHD criteria. There are 5 symptoms that are scored based on two items in the questionnaire because these symptoms contain double statements in the DSM-IV-TR criteria. For example, the symptom ‘often fails to give close attention to details or makes careless mistakes in schoolwork, work or other activities’, is referred to in the questionnaire by two questions ‘fail to give close attention to details in work’ and ‘make careless mistakes in work’. The 18 ADHD criteria consist of 9 symptoms related to the inattentive (IA) symptom domain and 9 symptoms to the hyperactive/impulsive (HI) symptom domain. A symptom was considered to be present whenever the answer ‘often’ or ‘very often’ was given. In this way, the variables IA-symptoms (range 0–9), HI-symptoms (range 0–9) and total ADHD symptoms (range 0–18) were derived.

### Imaging

Subjects were scanned at 1.5 Tesla (n = 302) and 3 Tesla (n = 350) MRI scanners and T1-weighted structural magnetic resonance imaging data (3D MPRAGE) were acquired (more information on the image acquisition can be found in Text S1). All scans covered the entire brain and had a voxel-size of 1×1×1 mm^3^. To calculate total brain volume, raw DICOM MR imaging data were converted to NIFTI format using the conversion as implemented in SPM5 (http://www.fil.ion.ucl.ac.uk/spm/software/spm5/). Normalizing, bias-correcting, and segmenting into gray matter, white matter, and cerebrospinal fluid was performed using the VBM toolbox (VBM5.1 Toolbox version 1.19) in SPM using priors (default settings). This method uses an optimized VBM protocol [18], [19] as well as a model based on Hidden Markov Random Fields (HMRF) developed to increase signal-to-noise ratio [20]. Total volume of gray matter, white matter, and cerebrospinal fluid was calculated by adding the resulting tissue probabilities. Total brain volume was defined as the sum of white matter and gray matter volume.

### Statistical analysis

The relation between self-reported ADHD symptoms and total brain volume was studied using linear regression analysis adjusting for age, gender and MRI field strength (1.5T or 3T). In order to explore effects of the distinct ADHD symptom domains on total brain volume, a similar analysis was performed including either Hyperactivity/Impulsivity (HI) symptom count or Inattentive (IA) symptom count as an independent variable. Permutation tests were performed to overcome multiple testing problems (see Text S1). As a downstream analysis, the association between ADHD symptom count and gray matter volume (correcting for white matter volume) and white matter volume (correction for gray matter volume) were performed.

In addition, we tested whether total brain volume of subjects with 6 or more self-reported ADHD symptoms in either one symptom domain or 6 or more symptoms in both domains (the equivalent of the cut-off for an ADHD diagnosis according to the DSM-IV-TR), differed from that of subjects with 4–5 self-reported ADHD (IA and/or HI) symptoms (4 being the threshold for increased impairment [17]) and/or subjects with 3 or less self-reported ADHD symptoms (IA and/or HI). Linear regression analysis was performed to assess the effect of this stratification on total brain volume, including age, gender and field strength as covariates. Individual groups were compared in post-hoc analyses. Finally, voxel-based morphometry (VBM) was performed to assess potential localized differences between these groups (for details see Text S1).

## Results

The demographics of the participants are displayed in Table 1. Overall, 37 subjects (5.6%) reported 6 or more current HI- or IA-symptoms in our study, where 6 would be the threshold for a DSM-IV-TR diagnosis of ADHD (for frequency tables of the ADHD symptom counts see Figure S1).

**Table 1:** Demographics of the study sample (n = 652).

**Age (mean ± SD; range)**	22.5±3.2 years (range 18–35)
**Male**	38.0%
**Education**	
**High school degree**	17.1%
**Bachelor student**	12.8%
**Master or PhD student**	70.1%
**ADHD symptoms (mean ± SD)**	2.8±2.7

Subjects were healthy individuals based on self-report.

### Association of Total Brain Volume and ADHD symptoms

**Figure 1:**
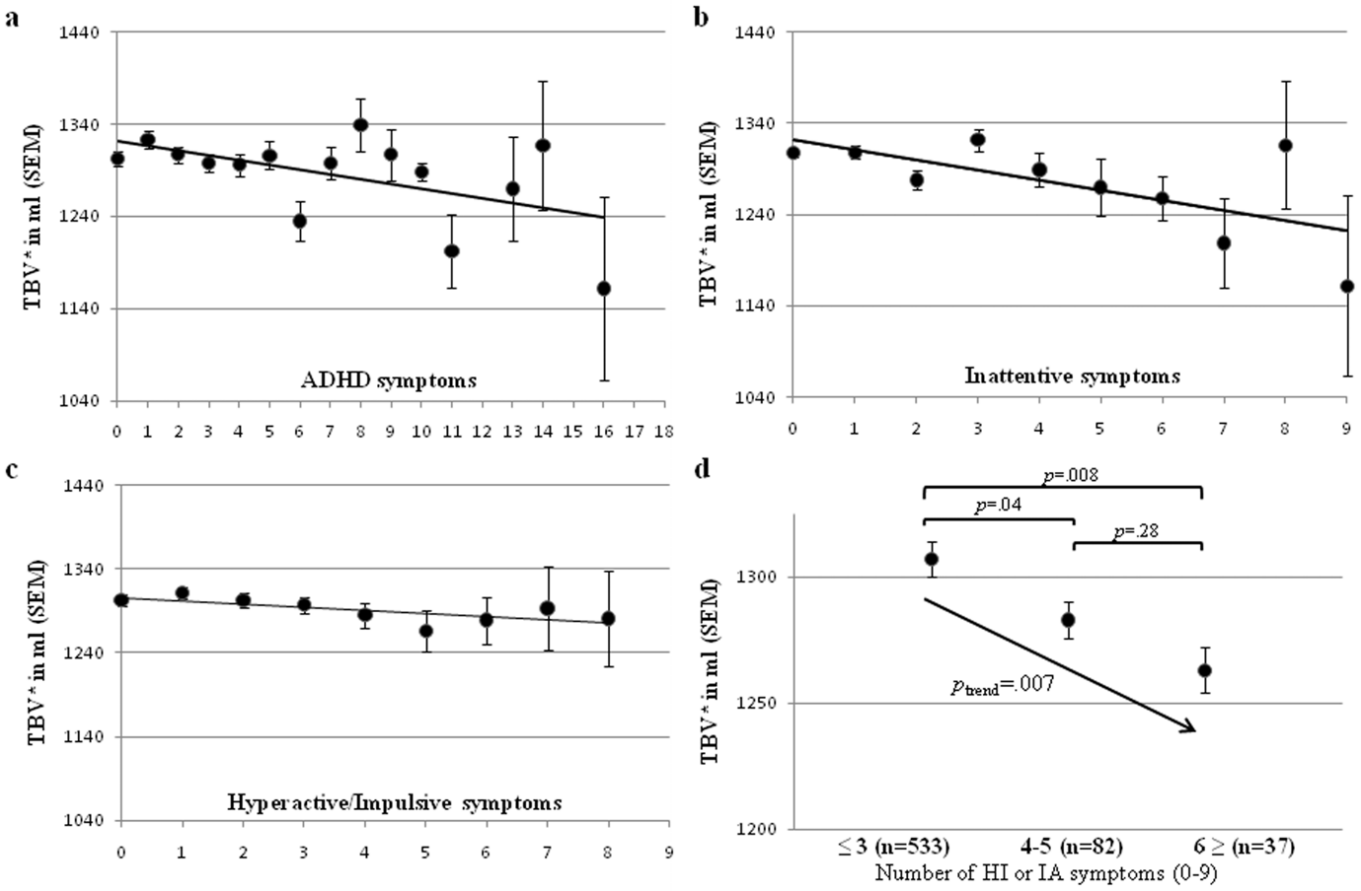
Distribution of total brain volume (TBV) and adult self-reported ADHD symptoms in a healthy sample (n = 652). (**a**) TBV is associated with the total number of self-reported ADHD symptoms. Of the two ADHD symptom domains, mainly the attentive domain contributes to TBV: (**b**) inattentive symptoms (β = −.09, *p*_nominal_ = .007, *p*_empirical_ = .013), and (**c**) hyperactive/impulsive symptoms (β = −.06, *p*_nominal_ = .064, *p*_emperical_ = .123). (**d**) Total brain volume across groups based on the number of self-reported ADHD symptoms. The group with 3 or less self-reported symptoms differed from the intermediate group and from the group with a number of ADHD symptoms corresponding to the ADHD diagnosis (≥6 in either of the two domains). *Total brain volume was adjusted for age, gender and field strength.

The total number of self-reported ADHD symptoms was significantly associated with total brain volume (β = −.08, nominal *p* = .007, experimental *p* = .014). The regression coefficients of the total number of self-reported ADHD symptoms were similar in the two independent samples scanned at different MRI field strengths (1.5T: β = −.07; 3T: β = −.09). Brain volume decreased with an increasing number of self-reported ADHD symptoms (Figure 1a). Total brain volume variation was mainly explained by IA-symptoms (β = −.09, *p*_nominal_ = .007, *p*_empirical_ = .013), rather than by HI- symptoms (β = −.06, *p*_nominal_ = .064, *p*_emperical_ = .123) (Figure 1b and 1c). The association was not explained by education level (*p* = .26). Neither total gray or white matter volume were associated with the number of self-reported ADHD symptoms (gray matter β = −.002, *p*_nominal_ = .92, *p*_emperical_ = .10, white matter β = −.04, *p*_nominal_ = .05, *p*_emperical_ = .99).

Grouping of individuals based on DSM-IV-TR criteria (≥6 self-reported symptoms, n = 37 more details in Figure S1) and known impairment threshold (4–5 self-reported symptoms, n = 82) [17] and comparing them to subjects with ≤3 self-reported symptoms (n = 533) showed significant differences between groups (*p* = .008 and *p* = .04, respectively), indicating that the threshold for brain volume reduction coincides with the threshold for functional impairment in patients (Figure 1d).

There were no significant differences in the local gray or white matter volumes assessed by the VBM analysis (both *p*(cluster)>0.05, data not shown) in our large sample. Indicating that brain volume was globally affected.

## Discussion

This study shows (a) that current ADHD symptoms in healthy adults, assessed by self-report as a continuum in the population and therefore largely independent of disease or treatment, has a neurobiological substrate; reduced total brain volume, (b) that this substrate is global rather than localized in the brain, and (c) that the threshold for impairment coincides with the threshold for brain volume reduction in patients.

The results of this study match the results of a previous study in healthy children and adolescents showing cortical thinning, not corrected for total brain volume, to be associated with symptoms of ADHD [12], and therefore supporting a dimensional aspect of biological substrates of ADHD.

Total brain volume reductions were global rather than localized in the brain. This suggests general mechanisms affecting brain development in neuron number, number of neurites and/or neuronal connections. This is also consistent with hypotheses based on findings from genome-wide association studies in ADHD implicating neuronal migration and neurite outgrowth in disease etiology [14], [21].

This study shows that self-reported inattention symptoms are the main predictor of total brain volume reductions. This could be explained by the known association between inattention and processing speed [22] and associations between the latter and brain volume reductions [23]. However, this study was not designed to study causative effects of brain volume reductions. Therefore, future studies with a better suited design would be necessary to provide additional information.

While this is the largest study of its kind and also the first to show neurobiological dimensionality of self-reported ADHD symptoms in an adult population, it also has a number of limitations. Firstly, we used a self-report measure of adult ADHD symptoms. Self-report measures are likely to be less accurate than interviews, but for the ADHD rating scale we know from research in patients that it has a substantial correlation with diagnostic assessment by a clinician [24]. A second potential limitation is the unavailability of other psychiatric symptom ratings to look at the specificity of our results. Although this was not our prime objective, it would be very informative to find out if symptoms of other psychiatric disorders are also associated with total brain volume or whether this association is specific for ADHD symptoms. Based on the study findings, suggesting a stronger role for inattentive symptoms one might hypothesize that depression and other disorders featuring such symptoms might also affect brain size. Thirdly, intelligence might be an important factor for total brain volume studies, since some studies have found moderate correlations between intelligence and total brain volume [25]. In the current study, direct intelligence measures were unavailable, but we used education level as a proxy. Education did not significantly contribute to the association between total brain volume and self-reported ADHD symptoms in this study. In addition, our entire sample is highly educated (mainly university students), which probably results in low variance in IQ measures. It is therefore not likely that IQ measures would change our findings.

Finding genes for ADHD has been proven difficult, our findings, in combination with those of previous studies showing brain volume to be heritable [15], indicate that total brain volume may be a target intermediate phenotype for future genetics studies directed at the identification of yet unknown ADHD risk genes.

## Supporting Information

Figure S1 **a**) Displayed are the number of subjects in this study (frequency) with their numbers of total self-reported ADHD symptoms (upper panel) and numbers of self-reported Inattentive symptoms and Hyperactive/Impulsive symptoms (lower panel). **b**) Displayed are subjects with 6 or more self-reported symptoms in both domains (upper bar), the number of subjects with 6 or more self-reported HI symptoms but less than 6 symptoms in the IA domain (middle bar) and the number of subjects with 6 or more self-reported IA symptoms but less than 6 symptoms in the HI domain (bottom bar).
(DOC)

Text S1 **Supplementary Methods.**
(DOC)
