# Supplementary material for: Current Self-Reported Symptoms of Attention Deficit/Hyperactivity Disorder Are Associated with Total Brain Volume in Healthy Adults
Source: PLoS One. 2012 Feb 10;7(2):e31273. doi: 10.1371/journal.pone.0031273 (PMC3277496; doi:10.1371/journal.pone.0031273)
Supplement: Figure S1 — a) Displayed are the number of subjects in this study (frequency) with their numbers of total self-reported ADHD symptoms (upper panel) and numbers of self-reported Inattentive symptoms and Hyperactive/Impulsive symptoms (lower panel). b) Displayed are subjects with 6 or more self-reported symptoms in both domains (upper bar), the number of subjects with 6 or more self-reported HI symptoms but less than 6 symptoms in the IA domain (middle bar) and the number of subjects with 6 or more self-reported IA symptoms but less than 6 symptoms in the HI domain (bottom bar). (DOC) [file pone.0031273.s001.doc]

**Figure S1 Self-reported ADHD symptoms in an adult population (n=652)**


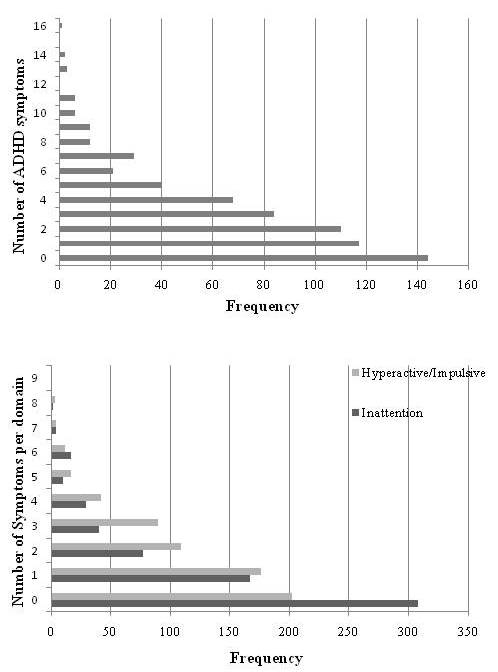


a


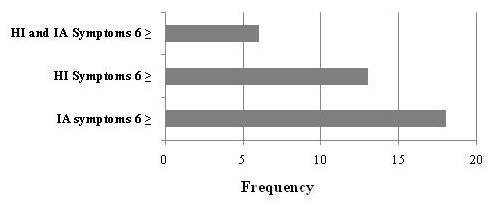


b

**Figure S1 a)** Displayed are the number of subjects in this study (frequency) with their numbers of total self-reported ADHD symptoms (upper panel) and numbers of self-reported Inattentive symptoms and Hyperactive/Impulsive symptoms (lower panel). **b)** Displayed are subjects with 6 or more self-reported symptoms in both domains (upper bar), the number of subjects with 6 or more self-reported HI symptoms but less than 6 symptoms in the IA domain (middle bar) and the number of subjects with 6 or more self-reported IA symptoms but less than 6 symptoms in the HI domain (bottom bar).
