# Supplementary material for: Current Self-Reported Symptoms of Attention Deficit/Hyperactivity Disorder Are Associated with Total Brain Volume in Healthy Adults
Source: PLoS One. 2012 Feb 10;7(2):e31273. doi: 10.1371/journal.pone.0031273 (PMC3277496; doi:10.1371/journal.pone.0031273)
Supplement: Text S1 — Supplementary Methods. (DOC) [file pone.0031273.s002.doc]

**S1 Supplementary Methods**

*Image acquisition*

*1.5T:* All images were acquired at 1.5T Siemens Sonata and Avanto scanners (Siemens, Erlangen, Germany), using small variations to a standard T1-weighted 3D-MPRAGE sequence (TR 2300 ms, TI 1100 ms, TE 3.03 ms, 192 sagittal slices, field of view 256 mm). These variations included a TR/TI/TE/slices of 2730/1000/2.95/176, 2250/850/2.95/176, 2250/850/3.93/176, 2250/850/3.68/176, and the use of GRAPPA parallel imaging with an acceleration factor of 2. All scans covered the entire brain and had a voxel size of 1x1x1 mm^3^. *3T:* All images were acquired at 3T Siemens Trio and TrioTim scanners (Siemens, Erlangen, Germany), using small variations to a standard T1-weighted 3D MPRAGE sequence (TR 2300 ms, TI 1100 ms, TE 3.93 ms, 192 sagittal slices, field of view 256 mm). These variations included a TR/TI/TE/slices of 2300/1100/3.03/192, 2300/1100/2.92/192, 2300/1100/2.96/192, 2300/1100/2.99/192, 1940/1100/3.93/176, 1960/1100/4.58/176, and the use of GRAPPA parallel imaging with an acceleration factor of 2.

*Permutation Analysis*

Analyses were performed using TBV and three different ADHD scores (Total number of ADHD symptoms, IA-symptoms and HI-symptoms) as dependents (covariates). In order to account for correlations among the phenotype and among the ADHD scores, permutations were performed to assess the experimental p-value. Permutations were performed by randomly reordering the records of ADHD scores of subjects over the records of TBV and covariates of subjects, leaving intact the link of covariates (age, gender, field strength) with TBV, and the correlation structure among the ADHD scores. Permutations were repeated 10,000 times. Within each permutated data set, all the models were refitted and p-values for association of the ADHD scores with TBV were obtained. Finally, the experimental p-value for a given nominal p-value was obtained by counting in how many permutations one or more of the analyses exceeded the nominal p-value. When analyses are independent there is a higher chance for one or more analyses within a permutation to randomly exceed a certain p-level, than when analyses are dependent.

*Voxel-Based Morphometry*

*Preprocessing:* Diffeomorphic image registration was performed using the DARTEL toolbox in SPM[1]. First, all images were realigned to templates created from 556 in-house datasets. Second, Jacobian scaled ('modulated') images were calculated and subsequently transformed to MNI space using affine transformation. Finally, all data were smoothed with an 8 mm FWHM Gaussian smoothing kernel.

*Analysis:* Data analysis was performed in SPM using the gray and white matter images. After grouping all data sets into 3 groups of ADHD symptoms (≥6 symptoms, 4-5 symptoms, and ≤3 symptoms), images with poor quality or artifacts were identified using the outlier analysis routine implemented in the VBM toolbox. Images that showed a deviation of more than 1.5 times the interquartile range from the median were discarded from further analysis. In our analysis, there were 11 subjects excluded from the VBM analysis for gray matter, and 17 for the VBM on white matter. These subjects did not differ from the subjects in the analysis on the number of ADHD symptoms [GM: p=.45, WM: p=.69].

Statistical analysis was performed using a GLM approach in SPM. A full-factorial ANCOVA was applied using ADHD symptom group. The participants' age, sex, total brain volume, and MRI scanner protocol were added to the model as covariates. F-tests were performed assessing the differences between the ADHD symptom groups. Cluster statistics were corrected for non-stationarity. Results were considered significant at *p*(uncorrected)<0.001 with a subsequent cluster statistics threshold *p*(cluster)<0.05.

REFERENCES

1. Ashburner J (2007) A fast diffeomorphic image registration algorithm. Neuroimage 38: 95-113.
